# Electronically Tunable Quadrature Oscillator Using Grounded Components with Current and Voltage Outputs

**DOI:** 10.1155/2014/572165

**Published:** 2014-07-10

**Authors:** Hua-Pin Chen

**Affiliations:** Department of Electronic Engineering, Ming Chi University of Technology, New Taipei, Taiwan

## Abstract

The electronically tunable quadrature oscillator using a single multiple-output current controlled current differencing transconductance amplifier (MO-CCCDTA) and grounded passive components is presented. The proposed configuration uses a single MO-CCCDTA, two grounded capacitors and one grounded resistor. Two high-output impedance quadrature current signals and two quadrature voltage signals with 90° phase difference. The oscillation condition and oscillation frequency of the proposed quadrature oscillator are independently controllable. The use of only grounded passive components makes the proposed circuit ideal for integrated circuit implementation.

## 1. Introduction

At present, there is a growing interest in designing analogue current-mode signal-processing circuits. Various new current-mode active building blocks have received considerable attentions owing to their larger dynamic range, greater linearity, wider bandwidth, and low power consumption with respect to operational amplifier-based circuits [1–6]. As a result, current-mode active components have been increasingly used to realize active filters and sinusoidal oscillators. Single-element-controlled sinusoidal oscillators find numerous applications in communication, control systems, signal processing, instrumentation, and measurement systems as test oscillators or signal generators [7–11]. A quadrature oscillator is used because the circuit simultaneously provides two sinusoids with 90° phase difference for a variety of applications, such as in telecommunications for quadrature mixers, in single-sideband generators, and in direct-conversion receivers or for measurement purposes in vector generators or selective voltmeters [11]. Quadrature signals are an essential part of modern RF-communication architectures such as zero-IF and image-reject receivers [12]. The quadrature oscillator described in [13] was developed for its possible use within a soil-impedance measurement system.

In 2003, a new current-mode active element that is called current differencing transconductance amplifier (CDTA) was introduced [14]. It has a pair of low-impedance current inputs *p* and *n* and an auxiliary terminal *Z*, whose outgoing current is the difference of input currents [15]. Owing to the current conveying, the CDTA is one of the modifications of the current conveyor. This element, connected with some external passive components, makes it possible to build many important applications like filters [16, 17] and oscillators [18, 19]. Many modifications of CDTA structure can be found in the literature. For example, duplication of the *Z* terminal is discussed in [20, 21], replacement of the transconductance amplifier with the voltage follower is discussed in [22], and replacement of the current differencing stage with the current buffer is discussed in [23]. In 2011, a new kind of resistorless voltage-mode and current-mode quadrature sinusoidal oscillator using a single differential voltage current-controlled conveyor transconductance amplifier (DVCCTA) and two grounded capacitors has been proposed [24]. The circuit provides two explicit quadrature current outputs and two quadrature voltage outputs, simultaneously. The oscillation condition and oscillation frequency are independently controllable. However, no voltage-mode and current-mode quadrature oscillator circuit based on single multiple-output current controlled current differencing transconductance amplifier (MO-CCCDTA) has been proposed which simultaneously provides two explicit quadrature current outputs and two quadrature voltage outputs in the same configuration. Recently, an electronically controllable current-mode quadrature oscillator using a single MO-CCCDTA as the active element has been introduced [25], in which the operational transconductance amplifiers (OTAs) at the two output ports can be controlled by an external bias current. This proposed circuit employs a single MO-CCCDTA, two grounded capacitors, and one grounded resistor and offers the advantages of (i) independent control of condition of oscillation (CO) and frequency of oscillation (FO) and (ii) low active and passive sensitivities. However, one of the current outputs is available on a grounded capacitor. One requires additional current follower for sensing and taking out the quadrature current output therein. The use of additional current follower with the virtual grounded input may result in floating capacitor realization for what is originally described as grounded capacitor realization.

In this paper, the author also proposes another simple electronically controllable grounded capacitor quadrature oscillator using a single MO-CCCDTA. The proposed circuit has all of the advantages by Prasad et al. [25] in addition to one more advantage of high-output impedance current outputs without using additional current followers. Both current-mode and voltage-mode quadrature signals can be simultaneously obtained in the proposed circuit. Sinusoidal oscillators which produce both current and voltage output signals are useful for their versatility. Since the proposed circuit consists of single MO-CCCDTA and all grounded passive components, it is more suitable for integrated circuit implementation.

## 2. Proposed Current-Mode and Voltage-Mode Quadrature Oscillator

**Figure 1:**
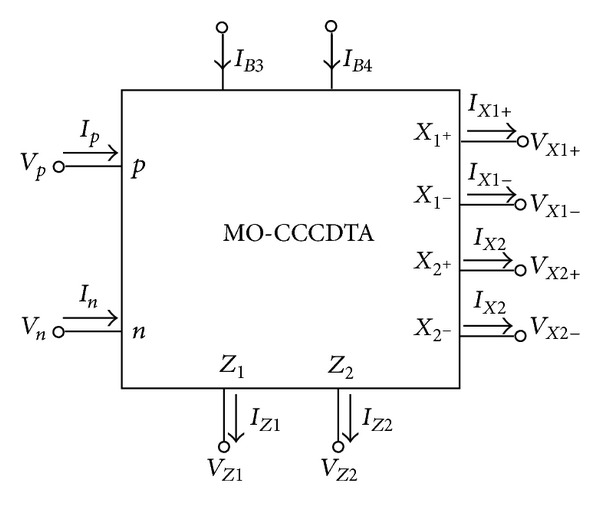
MO-CCCDTA symbol [25].

**Figure 2:**
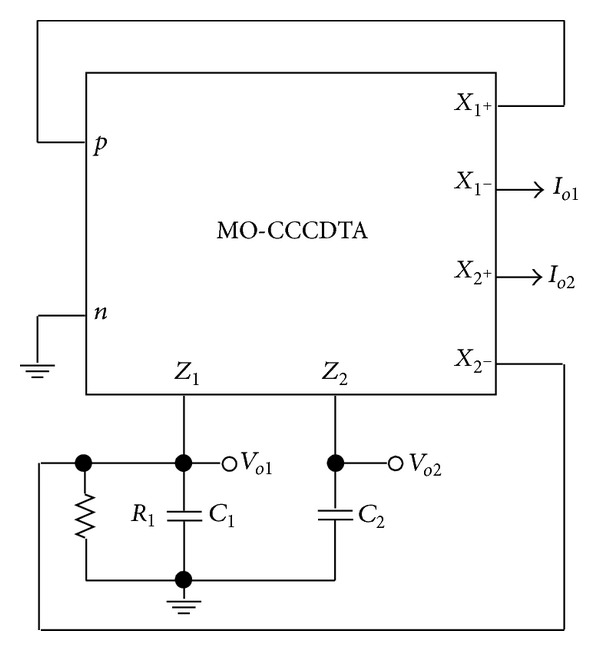
The proposed current-mode and voltage-mode quadrature oscillator.

MO-CCCDTA is relatively new active element [25] and has received considerable attention as current-mode active element. The MO-CCCDTA design concept originated from the CDTA [14]. The circuit symbol of the MO-CCCDTA is shown in Figure 1 [25]. It consists of two well-known and mutually independent building blocks, namely, *Z* copy CDTA and dual-output OTA. OTA is relatively independent. The MO-CCCDTA with multiple *Z* and *X* terminals has been used to create the quadrature oscillator. The terminal characteristics of the MO-CCCDTA are given by *V*_*p*_ = *V*_*n*_ = 0, *I*_*Z*1_ = *I*_*Z*2_ = *I*_*p*_ − *I*_*n*_, *I*_*X*1+_ = *g*_*m*1_ *V*_*Z*1_, *I*_*X*1−_ = −*g*_*m*1_ *V*_*Z*1_, *I*_*X*2+_ = *g*_*m*2_ *V*_*Z*2_, and *I*_*X*2−_ = − *g*_*m*2_ *V*_*Z*2_, where *V*_*z*_ = *I*_*Z*_ *Z*_*z*_ and *Z*_*z*_ is the external impedance connected to the *Z* terminal of the CDTA [25]. *I*_*B*3_ and *I*_*B*4_ indicated in Figure 1 show the external bias currents which control the transconductances to make the circuit electronically controllable [25]. The proposed current-mode and voltage-mode quadrature oscillator is shown in Figure 2. It is based on a single MO-CCCDTA, two grounded capacitors, and one grounded resistor. The use of only grounded passive components makes the proposed circuit ideal for integrated circuit implementation. Routine analysis of the proposed oscillator circuit of Figure 2 yields the following characteristic equation:

(1) $$\begin{array}{c} D\left(s\right)=s^{2}C_{1}C_{2}R_{1}+sC_{2}\left(1-g_{m1}R_{1}\right)+g_{m1}g_{m2}R_{1}. \end{array}$$

From (1), the CO is

(2) $$\begin{array}{c} g_{m1}R_{1}=1 \end{array}$$

and the FO is

(3) $$\begin{array}{c} f_{o}=\frac{1}{2\pi }\sqrt{\frac{g_{m1}g_{m2}}{C_{1}C_{2}}}. \end{array}$$

As indicated by (2) and (3), the CO can be controlled independently of FO by changing *R*_1_; the FO can be controlled by *g*_*m*2_ and hence it is current controllable by bias current *I*_*B*4_. From Figure 2, under steady state, the relationships between output currents *I*_*o*1_ and *I*_*o*2_ are

(4) $$\begin{array}{c} I_{o1}=\frac{\omega_{o}C_{1}}{g_{m2}}e^{-j90^{\circ }}I_{o2} \end{array}$$

ensuring the currents *I*_*o*2_ and *I*_*o*1_ to be in quadrature.

The relationships between output voltages *V*_*o*1_ and *V*_*o*2_ are

(5) $$\begin{array}{c} V_{o1}=\frac{\omega_{o}C_{2}}{g_{m1}}e^{j90^{\circ }}V_{o2} \end{array}$$

ensuring the voltages  *V*_*o*2_ and *V*_*o*1_ to be in quadrature.

Clearly, the current-mode and voltage-mode quadrature signals can be simultaneously obtained from Figure 2. Because the output impedances of the currents *I*_*o*1_ and *I*_*o*2_ in Figure 2 are very high, the two output terminals, *I*_*o*1_ and *I*_*o*2_, can be directly connected to the next stage. It should be noted that the two quadrature voltages are to be buffered before use. This would require larger area on the chip and more power consumption. To the best of author's knowledge, no single active element oscillator is proposed till date that simultaneously provides explicit quadrature current outputs and buffered voltage outputs, without using external voltage buffers.

## 3. Nonideality Analysis and Design Considerations

Taking the nonidealities of the MO-CCCDTA into account, the relationship of the terminal voltages and currents can be rewritten as *I*_*Zi*_ = *α*_*pi*_ *I*_*p*_ − *α*_*ni*_ *I*_*n*_ for *i* = 1, 2, *V*_*p*_ = *V*_*n*_ = 0, *I*_*X*1+_ = *β*_1_ *g*_*m*1_ *V*_*Z*1_, *I*_*X*1−_ = −*η*_1_ *g*_*m*1_ *V*_*Z*1_, *I*_*X*2+_ = *β*_2_ *g*_*m*2_ *V*_*Z*2_, and *I*_*X*2−_ = − *η*_2_ *g*_*m*2_ *V*_*Z*2_. *α*_*pi*_ represents the nonideal current transfer gain from the *p* terminal to the *Z*_*i*_ terminal of the MO-CCCDTA, *α*_*ni*_ denotes the nonideal current transfer gain from the *n* terminal to the *Z*_*i*_ terminal of the MO-CCCDTA, *β*_*i*_ is the transconductance inaccuracy factor from the *Z*_*i*_ terminal to the *X*_*i*+_ terminal of the MO-CCCDTA, and *η*_*i*_ is the transconductance inaccuracy factor from the *Z*_*i*_ terminal to the *X*_*i*−_ terminal of the MO-CCCDTA. Considering them, the modified CO and FO are given as

(6) $$\begin{array}{c} \text{CO}:\alpha_{p1}\beta_{1}g_{m1}R_{1}=1,\\ \text{FO}:f_{o}=\frac{1}{2\pi }\sqrt{\frac{\alpha_{p2}\beta_{1}\eta_{2}g_{m1}g_{m2}}{C_{1}C_{2}}}. \end{array}$$

The active and passive sensitivities are obtained as

(7) $$\begin{array}{c} S_{\alpha_{p2},\beta_{1},\eta_{2}}^{f_{o}}=S_{g_{m1},g_{m2}}^{f_{o}}=-S_{C_{1},C_{2}}^{f_{o}}=\frac{1}{2}. \end{array}$$

The active and passive sensitivities remain less than unity and hence the circuit exhibits a satisfactory sensitivity performance.

**Figure 3:**
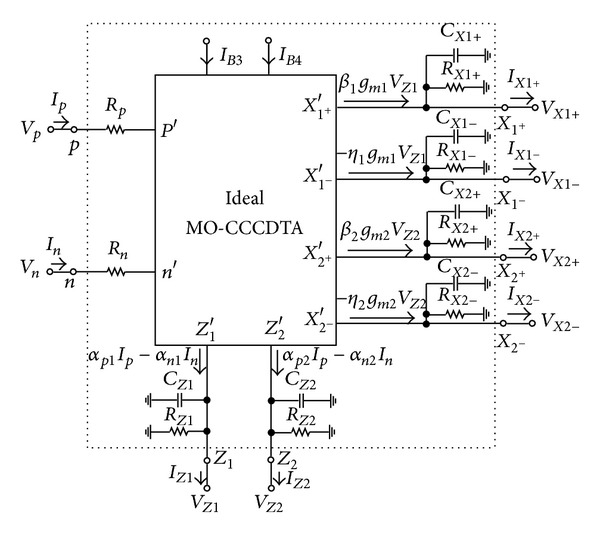
Nonideal equivalent circuit model of the MO-CCCDTA.

A study is next carried out on the effects of various parasitics of the MO-CCCDTA used in the proposed circuit. A practical MO-CCCDTA device can be modeled as ideal MO-CCCDTA with finite parasitic resistances and capacitances.  Figure 3 shows the nonideal MO-CCCDTA model including its parasitic elements. The nonzero parasitic input impedances at terminals *p* and *n* of the MO-CCCDTA are represented by *R*_*p*_ and *R*_*n*_, respectively. The parasitic resistance *R*_*Zi*_  (*i* = 1, 2) and parasitic capacitance *C*_*Zi*_ appear between the high-impedance *Z*_*i*_ terminals of the MO-CCCDTA and grounded. The parasitic resistance *R*_*Xi*_ and parasitic capacitance *C*_*Xi*_ appear between the high-impedance *X*_*i*_ terminals of the MO-CCCDTA and grounded. It is further noted that the proposed circuit employs external capacitors *C*_1_ and *C*_2_ parallel connecting to the terminals *Z*_1_ and *Z*_2_, respectively. As a result, the effects of the parasitic capacitances *C*_*Z*1_ and *C*_*Z*2_ can be absorbed, due to the fact that *C*_1_ ≫ *C*_*Z*1_, *C*_*X*2−_ and *C*_2_ ≫ *C*_*Z*2_. To alleviate the effects of parasitic impedance at terminal *X*_1+_, the MO-CCCDTA should be designed to have a very low input parasitic resistance *R*_*p*_ at terminal *p*. In the ideal case, the value of input parasitic resistance at terminal *p* is zero and terminal *p* is virtually grounded. Thus, the parasitic impedance at terminal *X*_1+_ is connected between a virtual grounded resistance *R*_*p*_ and a true grounded impedance (*R*_*X*1+_//*C*_*X*1+_). This fact affects the operating frequency in the high frequency region. To reduce its effect, one possible solution is to make the operating frequency *ω*_*o*_ ≪ 1/*C*_*X*1+_(*R*_*p*_//*R*_*X*1+_). The parasitic capacitance *C*_*Z*2_ can be absorbed in the external capacitance *C*_2_, but the presence of parasitic resistance at terminal *Z*_2_ would change the type of the impedance, which should be of a purely capacitive character. To alleviate the effects of the parasitic resistance *R*_*Z*2_, the operating frequency should be chosen such that *ω*_*o*_ ≫ 1/(*C*_2_ + *C*_*Z*2_)*R*_*Z*2_. In addition, the parasitic capacitance *C*_*X*1−_ (or *C*_*X*2+_) and parasitic resistance *R*_*X*1−_ (or *R*_*X*2+_) appear between the high-impedance *X*_1−_ (or *X*_2+_) terminal and ground. These parasitic components will affect the phase difference between the output currents. To reduce its effect, a possible solution is to make the operating frequency *ω*_*o*_ ≪ min⁡{1/(*C*_*X*1−_ *R*_*X*1−_), 1/(*C*_*X*2+_ *R*_*X*2+_)}. Therefore, the useful oscillation frequency range of the proposed oscillator is limited by the following conditions:

(8) 1(C2+CZ2)RZ2≪ωo≪min⁡{1CX1+(Rp//RX1+),1CX1−RX1−,1CX2+RX2+}.

Hence, the design procedure must satisfy the conditions *R*_*p*_≪*R*_*X*1+_, *C*_1_ ≫ *C*_*Z*1_ + *C*_*X*2−_, *R*_1_ ≫ *R*_*Z*1_//*R*_*X*2−_, and (8) to minimize the influence of the nonideal effects on the proposed circuit.

## 4. Simulation Results

**Figure 4:**
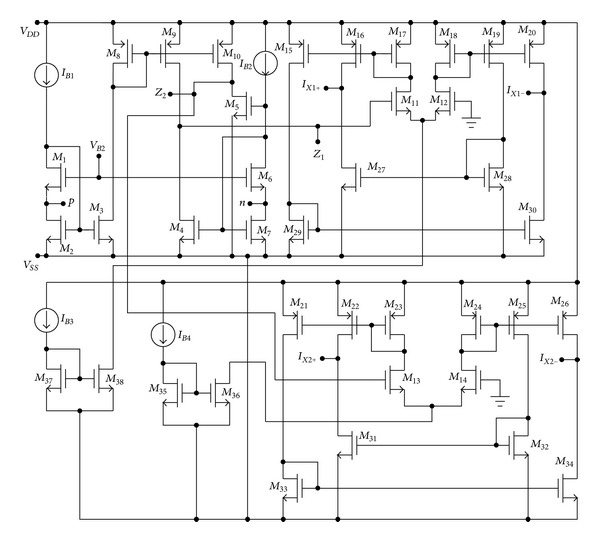
CMOS implementation of MO-CCCDTA [25].

**Figure 5:**
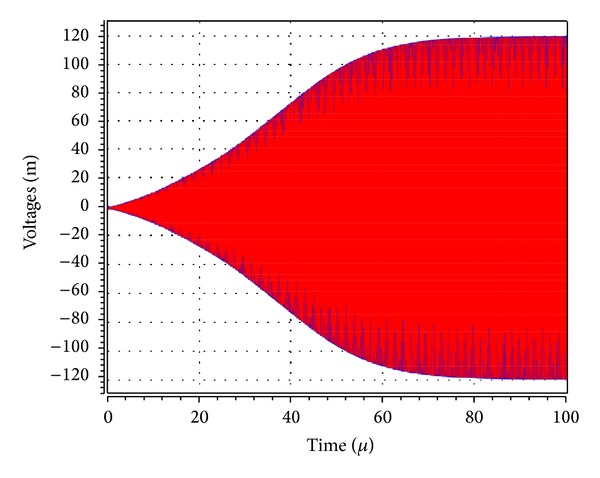
Transient amplitudes of the waveforms of *V*_*o*1_ (blue) and *V*_*o*2_ (red).

**Figure 6:**
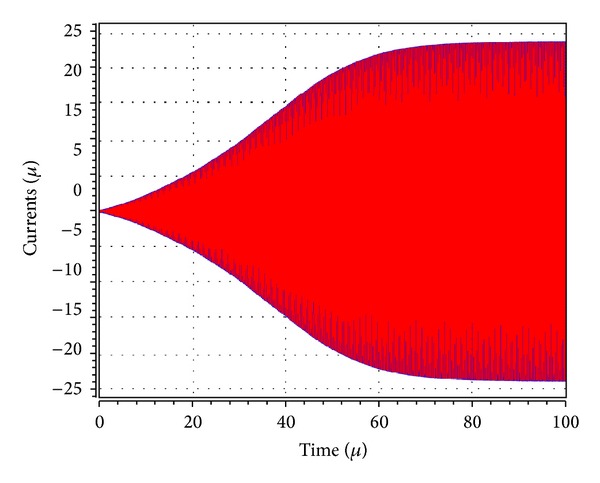
Transient amplitudes of the waveforms of *I*_*o*1_ (blue) and *I*_*o*2_ (red).

**Figure 7:**
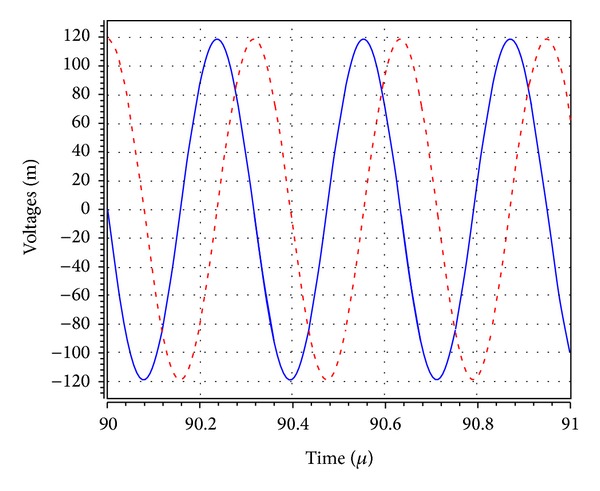
Simulated quadrature voltage output waves of *V*_*o*1_ (blue) and *V*_*o*2_ (red).

**Figure 8:**
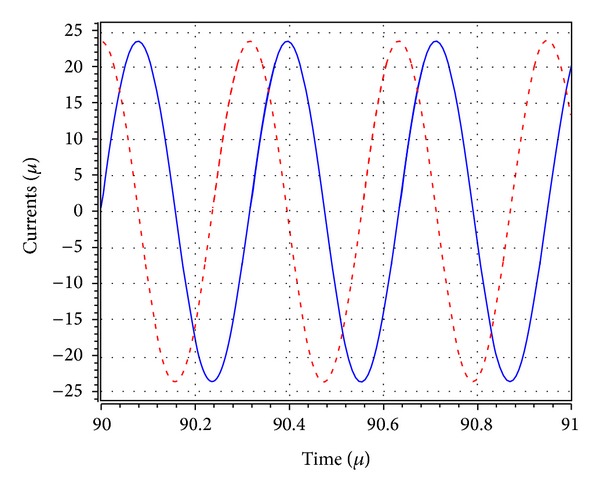
Simulated quadrature current output waves of *I*_*o*1_ (blue) and *I*_*o*2_ (red).

**Figure 9:**
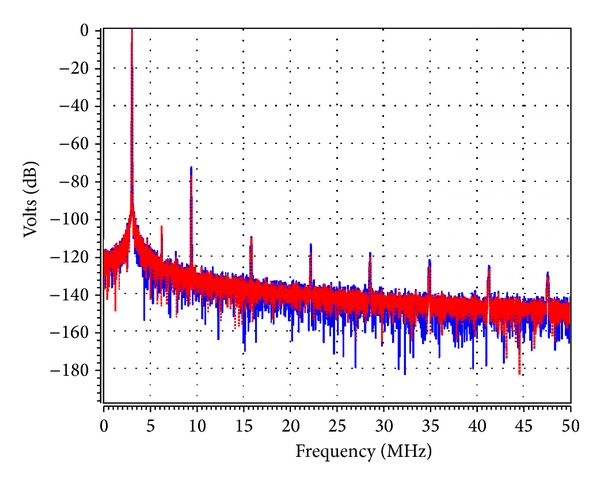
The simulated frequency spectrum of *V*_*o*1_ (blue) and *V*_*o*2_ (red).

**Figure 10:**
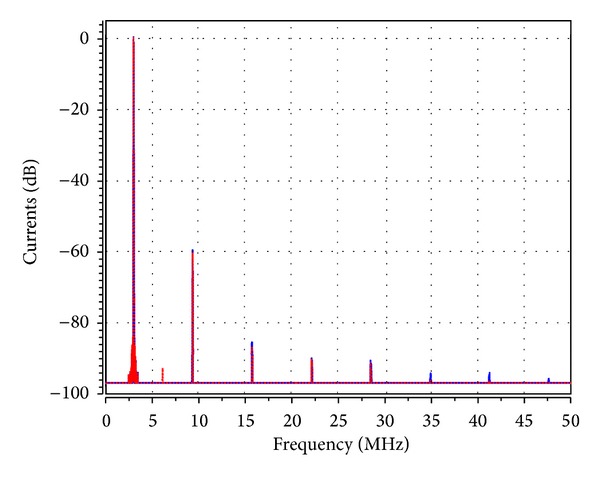
The simulated frequency spectrum of *I*_*o*1_ (blue) and *I*_*o*2_ (red).

**Figure 11:**
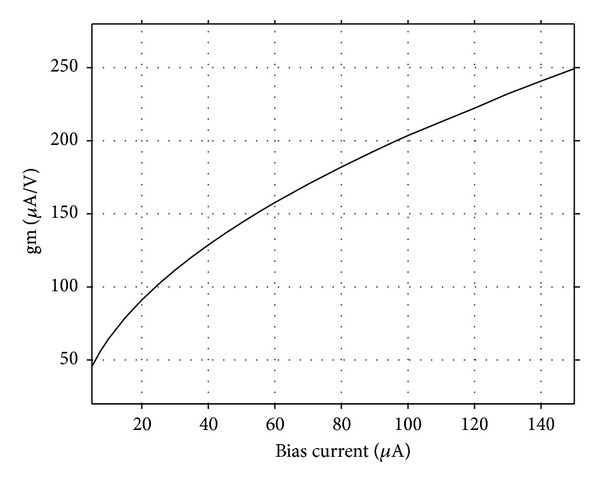
Variation of transconductance with bias current *I*_*B*4_.

**Figure 12:**
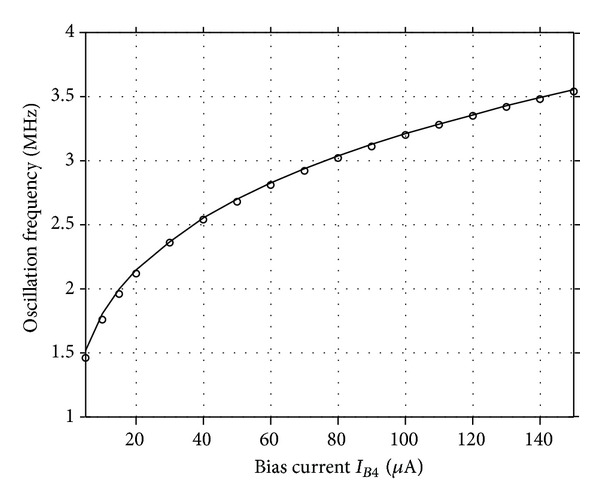
Oscillation frequency against the bias current *I*_*B*4_ of the circuit (o, simulated frequency, theoretical curves).

**Figure 13:**
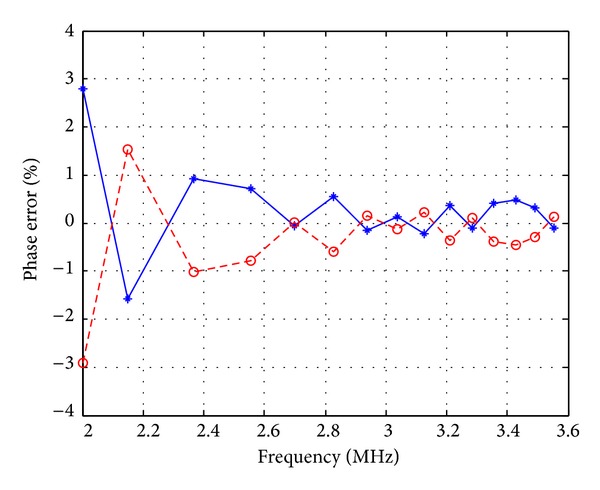
Phase error as a function of oscillation frequency (blue: phase error versus *V*_*o*1_ and *V*_*o*2_ and red: phase error versus *I*_*o*1_ and *I*_*o*2_).

**Figure 14:**
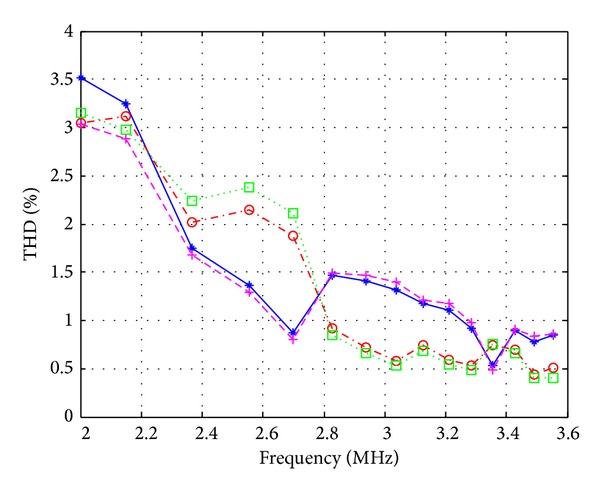
Total harmonic distortion (THD) as a function of oscillation frequency (blue: *V*_*o*1_; red: *V*_*o*2_; pink: *I*_*o*1_; and green: *I*_*o*2_).

In order to verify the theoretical analysis, the proposed oscillator has been simulated using HSPICE program by using TSMC 0.18 *µ*m CMOS process technology process parameters. The CMOS implementation of the MO-CCCDTA is shown in Figure 4 [25]. The aspect ratios (W/L) of the MOS transistors were taken as 8.75/0.18 for M1–M7; 17.5/0.18 for M8–M10; 10/0.5 for M11–M14; 25/0.8 for M15–M26; 8/0.8 for M27–M34; and 35/0.25 for M35–M38. It may be noted that some of the MOS transistors were used with large widths. This would occupy large area on the chip. The supply voltages are *V*_*DD*_ = −*V*_*SS*_ = 0.9 V; the biasing currents are *I*_*B*1_ = *I*_*B*2_ = 50 *μ*A and *I*_*B*3_ = *I*_*B*4_ = 96.5 *μ*A (*g*_*m*_≅200 *μ*S). *I*_*B*1_ and *I*_*B*2_ are the biasing currents for the device to perform the current differencing operation, while MO-CCCDTA transconductances are controlled by *I*_*B*3_ and *I*_*B*4_. The two capacitors in Figure 2 were set to be equal by *C*_1_ = *C*_2_ = 10 pF. *R*_1_ was adjusted to 5.02 kΩ to start the oscillations. The theoretical oscillation frequency using this design was 3.183 MHz. The startup output waveforms for both the quadrature voltages and currents were shown in Figures 5 and 6, respectively. The steady state output waveforms for both the quadrature voltages and currents were shown in Figures 7 and 8, respectively. The frequency spectrums for both the quadrature voltages and currents were shown in Figures 9 and 10, respectively. From the simulation results, the oscillation frequency of *f*_*o*_≅3.17 MHz is obtained, which agrees very well with the theoretical analysis. The total harmonic distortions for voltage and current outputs *V*_*o*1_, *V*_*o*2_, *I*_*o*1_, and *I*_*o*2_ are 1.02%, 0.91%, 1.01%, and 0.87%, respectively.  Figure 11 shows the variation of the transconductance value by changing *I*_*B*4_ from 5 to 150 *μ*A. The electronic tuning of the *V*_*o*1_ oscillation frequency with the bias current *I*_*B*4_ was shown in Figure 12. These simulations results are close to the theoretical prediction and confirm the feasibility of the proposed configuration. In addition, the effect of mismatch errors of the current mirror on the performance of the proposed circuit is investigated by setting the value of *I*_*B*4_ = 96.5 *μ*A with errors of −10%, −5%, +5%, and +10%, respectively. Simulation results show the slight oscillation frequencies which are approximately 3.09 MHz, 3.13 MHz, 3.21 MHz, and 3.25 MHz, respectively, all of which are less than ±3% in disagreement with the designed oscillation frequency of *f*_*o*_ = 3.183 MHz. Compared with the designed oscillation frequency, *f*_*o*_ = 3.183 MHz, the frequency deviation due to mismatch error of the current mirror is acceptable.  Figure 13 shows the phases of quadrature voltage and current outputs. In Figure 13, the output files of Fourier analysis from simulation results were used for calculating the phase error.  Figure 14 shows the total harmonic distortion values of voltage and current output signals. It can be seen that the total harmonic distortion values of output voltages and currents are less than 3.6%. The proposed sinusoidal oscillator has a simple topology and provides voltage-mode and current-mode operation with electronically tunable properties. The power dissipation is 1.427 mW.

## 5. Conclusion

In this paper, a new quadrature oscillator circuit using a single MO-CCCDTA, two grounded capacitors, and one grounded resistor is presented. The oscillation condition and oscillation frequency of the proposed quadrature oscillator have the advantage of being independently controllable. Two high-output impedance sinusoid currents with a 90° phase difference are available in the proposed configuration. The use of all grounded passive elements makes the proposed circuit ideal for integrated circuit implementation. Both current-mode and voltage-mode quadrature signals can be simultaneously obtained in the proposed circuit. HSPICE simulation results have confirmed the workability of the circuit.
